# Multiview Self-Supervised Segmentation for OARs Delineation in Radiotherapy

**DOI:** 10.1155/2021/8894222

**Published:** 2021-03-05

**Authors:** Cong Liu, Xiaofei Zhang, Wen Si, Xinye Ni

**Affiliations:** ^1^Faculty of Business Information, Shanghai Business School, Shanghai 200235, China; ^2^The Affiliated Changzhou No. 2 People's Hospital of Nanjing Medical University, Changzhou 213003, China; ^3^Center of Medical Physics, Nanjing Medical University, Changzhou 213003, China; ^4^Department of Comprehensive Treatment, Qingzhou Hospital of Traditional Chinese Medicine, Weifang 262500, China; ^5^Huashan Hospital, Fudan University, Shanghai 200031, China

## Abstract

Radiotherapy has become a common treatment option for head and neck (H&N) cancer, and organs at risk (OARs) need to be delineated to implement a high conformal dose distribution. Manual drawing of OARs is time consuming and inaccurate, so automatic drawing based on deep learning models has been proposed to accurately delineate the OARs. However, state-of-the-art performance usually requires a decent amount of delineation, but collecting pixel-level manual delineations is labor intensive and may not be necessary for representation learning. Encouraged by the recent progress in self-supervised learning, this study proposes and evaluates a novel multiview contrastive representation learning to boost the models from unlabelled data. The proposed learning architecture leverages three views of CTs (coronal, sagittal, and transverse plane) to collect positive and negative training samples. Specifically, a CT in 3D is first projected into three 2D views (coronal, sagittal, and transverse planes), then a convolutional neural network takes 3 views as inputs and outputs three individual representations in latent space, and finally, a contrastive loss is used to pull representation of different views of the same image closer (“positive pairs”) and push representations of views from different images (“negative pairs”) apart. To evaluate performance, we collected 220 CT images in H&N cancer patients. The experiment demonstrates that our method significantly improves quantitative performance over the state-of-the-art (from 83% to 86% in absolute Dice scores). Thus, our method provides a powerful and principled means to deal with the label-scarce problem.

## 1. Introduction

Radiotherapy is an important treatment option for many cancers, and the complex anatomy and distribution of normal organs in head and neck cancer may lead to damage of organs at risk (OARs), resulting in complications such as the oral mucosa damage, larynx edema, and dysphagia. To mitigate the toxic side effects of radiotherapy, modern radiotherapy techniques, such as intensity-modulated radiotherapy and volumetric-arc-modulated therapy, are capable of implementing highly conformal dose distribution for the target areas of tumors, reducing the radiation dose that endangers OARs, therefore reducing radiation-induced toxicity [1]. A key step in reducing the toxic effects of radiation exposure is the accurate delineation of OARs, which is usually performed manually by clinicians based on computed tomography (CT) scans and requires a great deal of time and effort. In the head and neck case, for example, many tumors are treated over a large area, covering a large number of OARs that have complex anatomical structures. Therefore, OARs delineations in head and neck cancer are time consuming and laborious to outline manually.

Traditional automatic delineation methods are mostly based on Atlas [2], with the drawbacks of the large computational burden and the reliance on Atlas templates. Recently, deep learning methods show their capability of learning anatomical features for delineation directly from the images without templates [3, 4]. Given enough delineation labels, a supervised deep learning model can produce clinically acceptable results. Usually, decent performance requires hundreds of labels. However, collecting manual delineations is expensive and hard to be scaled up. Considering the amount of unlabelled data is substantially more than a limited number of clinician curated labelled data, it is kind of wasteful not to use them. However, the unsupervised learning is very hard and usually works much less efficiently than supervised learning. Recently, self-supervised learning and contrastive learning have shown great promise, achieving state-of-the-art results [5, 6].

To address the label-scarce issue, a novel contrastive learning framework was developed and evaluated on a large-scale head and neck cancer dataset. Clinical validation of the accuracy and efficiency of the new method lays the foundation for its clinical application.

## 2. Methods

### 2.1. Multiview Contrastive Learning

**Figure 1:**
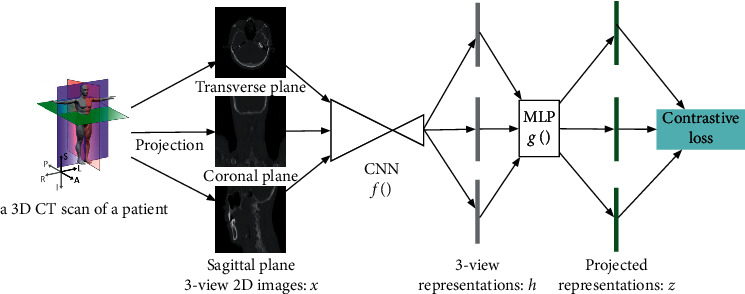
A overall illustration of the multiview self-supervised learning.

Inspired by recent contrastive learning algorithms [5, 6], this study proposes a novel method that learns representations by maximizing agreement between different views of the same patient via a contrastive loss in the latent space. As illustrated in Figure 1, this method comprised the 3D CT image is first projected into three 2D views (coronal, sagittal, and transverse planes), then an existing deep convolutional neural network is used to obtain the representations of the three views, and finally, a contrastive loss is used to pull representation of different views of the same image closer (“positive pairs”) and push representations of views from different images (“negative pairs”) apart.

As shown in Figure 1, a 3D CT scan is first projected to three correlated views of the same patient, denoted *x*_1_, *x*_2_, and *x*_3_, which are considered as the positive pair. A convolutional neural network- (CNN-) based feature encoder *f*(*x*) extracts representation vectors *h* from previous 2D images. For the easily adaption to the segmentation task, we choose the commonly used UNet [7]. Only the encoder part of UNet is used during this self-learning stage. The whole UNet is joint trained later during the full-supervised stage. A multiple-layer perceptron (MLP) *g*(*h*) projects representations to the space where contrastive loss is applied. An MLP with one hidden layer and batch normalization is used to obtain the projected *z*. Finally, a contrastive loss function is defined to distinguish between similar and dissimilar representations:

(1) $$\begin{array}{c} L_{i,j}=\frac{-\log\exp\left(z_{i}^{T}z_{j}/\tau \right)}{\sum_{k}1_{\left[k\neq i\right]\exp\left(z_{i}^{T}z_{j}/\tau \right)}}, \end{array}$$

where the contrastive loss is defined for a positive pair (*i*, *j*), 1_[*k* ≠ *i*]_ is the indicator function whose value equates to 1 if and only if *k* ≠ *i*, and *τ* is the temperature. The loss is computed across all positive pairs in a minibatch. Typical contrastive training relyies on large minibatch sizes such as 4098, but we avoid such hardware demanding setting by adopting the memory banks technology, which uses a slow-moving average network (momentum encoder) to maintain consistent representations of negative pairs drawn from a memory bank. Formally, denoting the parameters of query encoders *f*() and *g*() as *θ*_*q*_ and those of key encoders as *θ*_*k*_, we update *θ*_*k*_ as

(2) $$\begin{array}{c} \theta_{k}=m\theta_{k}+\left(1-m\right)\theta_{q}, \end{array}$$

where *m* is a momentum parameter that exponentially moving averages parameters. The network parameter *θ*_*q*_ is optimized as usual. The advantage of this design is that it provides a principle way to discriminate information from 3 views for the same patient to obtain the improved representations for a downstream segmentation task.

### 2.2. Data

We used two datasets in this study. Dataset 1 contains an in-house collection of 188 CT scans from Shanghai Huashan Hospital. We manually annotate 24 OARs for this dataset, which include the brain, brain stem, spinal cord, spinal cord cavity, left eyeball, right eyeball, left crystal, right crystal, left optic nerve, right optic nerve, optic nerve cross, pituitary gland, left parotid gland, right parotid gland, oral cavity, mandible, left mandibular joint, right mandibular joint, left temporal lobe, right temporal lobe, larynx, pharynx, trachea, and thyroid. The organs were divided into 4 categories based on their importance. Among them, organ class A distributes among many CT slices, and the automatic delineation can reduce the repetitive manual drawing; organ class B has few slices but is delineated more frequently; organ class C is used less for planning; and class D involves critical physiology functions but is smaller and has less time-consuming drawing. Dataset 2 consists of a CT scans Head-Neck Cetuximab (HNC) dataset, which is collected from The Cancer Imaging Archive (TCIA) which is publicly available [8]. HNC consists of 32 patients' data from a clinical trial for stage III and IV head and neck carcinomas. We followed the same procedure as described in generating dataset 1 to annotate OARs in each of the CT scans.

### 2.3. Experiment Organization

We first train the network with all available samples from two datasets in the proposed self-supervised way and then fine tune the network on the 150 labelled patients in dataset 1. The remaining 38 patients in dataset 1 and 32 patients in dataset 2 are used to evaluate the performance. Four NVIDIA TITAN 3090 GPUs and PyTorch [9] deep learning framework are used to develop codes. We implement the details suggested in literature [6] to boost the performance, i.e., LARS, cosine learning rate, and the MLP projection head. The initial learning rate is set to 0.001 for 60,0000 iterations during the unsupervised training stage, and the initial learning rate is set to 0.0001 for 5,000 iterations during the fine tuning stage.

### 2.4. Quality Evaluation Metrics

Dice coefficients and Hausdorff distances are used to quantify and analyze the accuracy of the automatic delineation. The Dice is used to evaluate the accuracy of the inner region of OARs, and Hausdorff is used to evaluate the accuracy of the OARs boundaries.

## 3. Results and Discussion

### 3.1. Contour Accuracy

In order to verify the quality of the new method's delineations, it is compared with the Ua-Net [3] and Anatomy-Net [4] methods. Ua-Net was published in Nature 2019 and is the current best deep learning-based method. Anatomy-Net is another deep learning method dedicated to OARs of head and neck cancer, published in Medical Physics in 2018. The Dice score of the three methods are reported in Table 1. As shown in the table, the accuracy of our method was better than the other methods for most OARs. The average Dice score of the three methods was 0.86, 0.83, and 0.80, respectively. Our method improved the accuracy by 3.5% over Ua-Net and by 6.5% over Anatomy-Net. Ua-Net outperformed our method on the brain stem, oral cavity, and trachea, which may be attributed to its 3D nature, which is advantageous for organs with a large transverse span.

**Figure 2:**
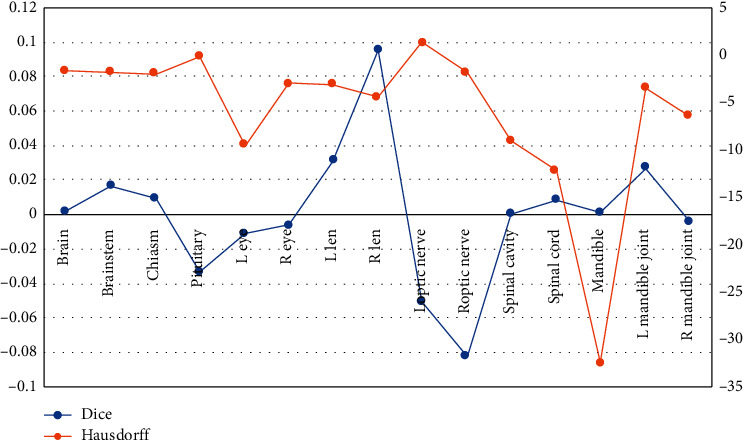
Quantitative comparison of our method with the supervised deep learning methods.

To validate the organ boundary accuracy of our method over the supervised deep learning method, Figure 2 reports the Dice difference and Hausdorff difference of the two methods. (a) Dice difference of >0 indicates that our method is superior, and a Hausdorff difference of <0 indicates that our method is superior. As shown in the figure, the Dice difference between the two methods is very small (Dice difference on the left vertical axis) with a mean value of 0.0001. However, the Hausdorff difference between the two methods is very large (Hausdorff difference on the right vertical axis) with a mean value of −5.96, indicating that our method has better organ boundary accuracy.

**Figure 3:**
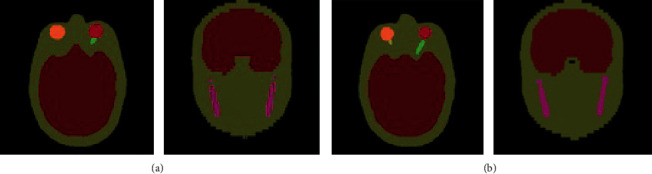
Qualitative comparison of our method with supervised deep learning methods. (a) Supervised deep learning methods. (b) Our method.

Figure 3 compares the delineation results of our method and the supervised deep learning method [7] on two sets of data. As seen in the figure, the delineation from the supervised method misses the optic nerves while our method delineates the optic nerves correctly. Similarly, the supervised method incorrectly predicts the chiasm, while our method correctly delineated this organ.

## 4. Conclusions

This study proposes and evaluates a novel deep-learning-based delineation method. Clinical evaluations show that our method has a delineation accuracy of 3.5% (Dice) and a boundary accuracy of 5.96 (Hausdorff) higher than the current best method. The advantage of our method is the integration of information from all three views of the CT to achieve better delineations than a single view.

This study has the following limitations. First, only CT images were used to delineate OARs. Some anatomical structures, such as crystals, have a low contrast on CT and are difficult to delineate with CT alone. Therefore, it is very important to integrate information from other modal images (e.g., MRI). Secondly, although delineation labels are defined by a senior physician, there will always be errors in manual delineation. Therefore, a standard delineation dataset is required in the future. One advantage of deep learning in this regard is that it ensures that the delineations are consistent across hospitals and individuals. Third, the number of delineation labels is still small, which limits the capacity of the deep network. There is a need to collect more standard delineation from more sources to improve the cross-domain adaptability and generalization of the deep network in the future.

In summary, a novel deep learning method is proposed in this study, which can delineate OARs in head and neck cancer, with better accuracy than the current state-of-the-art methods. The new method can save the clinician's manual delineation time and, thus, is clinically applicable and has the potential of clinical promotion.

## Figures and Tables

**Table 1:** Comparison of the proposed method with the state-of-the-art supervised methods (the higher the Dice, the better).

OARs	Our's	Ua-Net [3]	Anatomy-Net [4]
Brain	0.970	N/A	N/A
Brain stem	0.860	0.881	0.826
Spinal cord	0.862	0.856	0.803
Spinal cord cavity	0.891	N/A	N/A
Eye L	0.927	0.897	0.884
Eye R	0.927	0.919	0.892
Len L	0.801	0.793	0.772
Len R	0.821	0.746	0.78
Optical nerve L	0.798	0.693	0.725
Optical nerve R	0.750	0.718	0.729
Chiasm	0.770	0.618	0.605
Pituitary	0.724	N/A	N/A
Parotid L	0.837	0.839	0.822
Parotid R	0.872	0.847	0.822
Oral cavity	0.901	0.948	0.876
Mandible	0.921	0.925	0.919
Mandible joint L	0.873	0.824	0.816
Mandible joint R	0.865	0.837	0.817
Temporal lobe L	0.896	0.8478	0.866
Temporal lobe R	0.901	0.8413	0.857
Larynx	0.899	0.933	0.83
Pharynx	0.794	N/A	N/A
Trachea	0.866	0.812	0.793
Thyroid	0.857	0.827	0.718
*Mean*	*0.86*	*0.83*	*0.80*

## Data Availability

The CT data used to support the findings of this study have not been made available because of patient privacy.
